# Efficiency of NZ2114 on Superficial Pyoderma Infected with *Staphylococcus pseudintermedius*

**DOI:** 10.3390/ph17030277

**Published:** 2024-02-22

**Authors:** Na Yang, Yan Huang, Yuanyuan Li, Da Teng, Ruoyu Mao, Ya Hao, Lingyun Wei, Jianhua Wang

**Affiliations:** 1 Gene Engineering Laboratory, Feed Research Institute, Chinese Academy of Agricultural Sciences, Beijing 100081, China; nana_891230@126.com (N.Y.); hy118023@163.com (Y.H.); 82101202035@caas.cn (Y.L.); tengda@caas.cn (D.T.); maoruoyu@caas.cn (R.M.); haoya@caas.cn (Y.H.); 2 Innovative Team of Antimicrobial Peptides and Alternatives to Antibiotics, Feed Research Institute, Chinese Academy of Agricultural Sciences, Beijing 100081, China; 3 Key Laboratory of Feed Biotechnology, Ministry of Agriculture and Rural Affairs, Beijing 100081, China; 4 School of Environmental Ecology and Biological Engineering, Wuhan Institute of Technology—WIT, Wuhan 430075, China

**Keywords:** antimicrobial peptides, NZ2114, *Staphylococcus pseudintermedius*, transdermal delivery, chemical permeation enhancers, superficial pyoderma

## Abstract

*Staphylococcus pseudintermedius* (*S. pseudintermedius*) is the main pathogen causing pyoderma of canines. With the emergence of drug-resistant bacteria, traditional antibiotic treatments are limited. As a potential antibacterial agent, NZ2114 was effective against *S. pseudintermedius*, including drug-resistant strains. Its bactericidal efficacy was superior to mupiroxacin, ofloxacin and lincomycin. To facilitate the transcutaneous delivery of NZ2114 for the treatment of superficial pyoderma, chemical permeation enhancers were added since water-soluble NZ2114 does not easily penetrate the skin lipid layer. Two different NZ2114 sprays were prepared by combining 1% Azone + 10% propylene glycol (PG) or 5% N-methylpyrrolidone (NMP) + 10% PG with NZ2114 after screening. The cumulative permeability of NZ2114 sprays were 244.149 and 405.245 μg/cm^2^ at 24 h with an in vitro percutaneous assay of mice skin, which showed a 244% and 405% increase in skin permeability than NZ2114, respectively. In addition, the efficacy of NZ2114 sprays in reducing skin bacteria colonisation was demonstrated in a mouse model of superficial pyoderma (24 mice, 3 mice/group) induced by *S. pseudintermedius*, and the 5% NMP + 10% PG + NZ2114 group had the best therapeutic effect compared to the other groups. This preparation did not cause any skin irritation, laying the foundation for the development of an effective and non-toxic topical product.

## 1. Introduction

*Staphylococcus pseudintermedius* (*S. pseudintermedius*), as an opportunistic pathogen of canine [1], is a major cause of skin and ear infections. Canine pyoderma, a bacterial infection of the skin mainly caused by *S. pseudintermedius*, is one of the skin conditions frequently encountered in canines [2]. According to the depth of infection, pyoderma can be divided into surface infection, superficial infection and deep infection [3]. Superficial pyoderma, such as pustulosis and exfoliated superficial pyoderma, is characterized by papules, pustules and epidermal rings [4]. Humans are not natural colonizers of *S. pseudintermedius* but could also be pathogen carriers after contact with canines with *S. pseudintermedius* infection. As a result, *S. pseudintermedius* infection has become a major zoonotic public health problem [5]. Currently, the classical treatment for canine pyoderma is local or systematic application of antibiotics. The emergence of multidrug-resistant and methicillin-resistant *S. pseudintermedius* (MRSP) not only limits the therapeutic efficacy of clinical drugs against the bacterial infections but also leads to recurrent, difficult-to-treat canine pyoderma infections [6,7]. In addition, MRSP can be transmitted to humans and animals by various means, posing a serious threat to public health [1]. Clinical canine pyoderma is predominantly a complex infection and is often associated with skin tissue ulceration after a longer latency, leading to a prolonged course of the disease. The repair of skin structures occurs in a synchronized cycle of a length of at least 21 days, which can further prolong the duration and situation of the disease; this actual complexity adds to the difficulty of common drug therapy, and also it is very difficult to simulate with model animals, with artificial attack and treatment, with fresh wounds on intact healthy skin. Given the increasing resistance of the bacteria that causes pyoderma and the complexity of the disease, it is very necessary to find new more effective antimicrobial drugs besides classical antibiotics and chemical drugs to control bacterial pyoderma.

**Figure 1:**
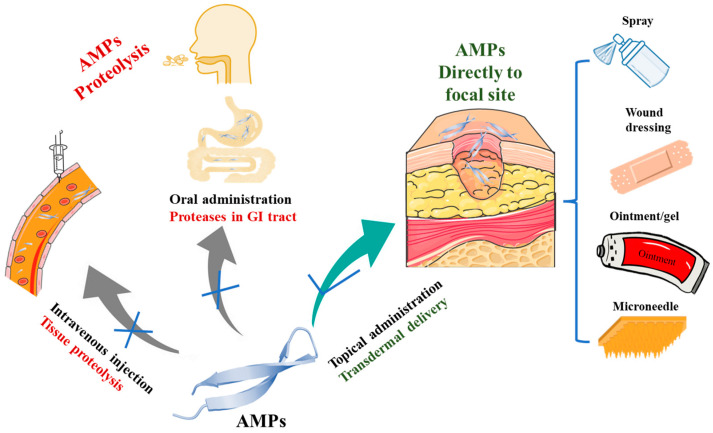
Effect of different administrations of AMPs on the therapeutic effect of dermatologic diseases. For injection and oral administration, the AMPs are hydrolyzed by tissue protease and GI tract protease in vivo, making it difficult to reach the focal site; for topical administration, AMPs can be delivered transdermally and act directly at the focal site. “×” indicates unsuitable mode of application, “√” indicates suitable mode of application.

Antimicrobial peptides (AMPs), a class of small molecular peptides that widely exist in nature, are a component of almost all innate biological defense systems [8], showing advantages of rapid killing, strong membrane penetration, high specificity and low resistance to pathogens compared to other small molecule drugs [9,10,11]. AMPs combine the advantages of vaccines’ specificity and antibiotics’ high activity in the iron triangle cooperation form, while avoiding their disadvantages of being energy expending and causing pathogen mutation, drug residues and resistance. This is due to the unique enzyme sensitivity of AMPs that results in their easy degradation, but it is also this property that limits their clinical translation to oral and intravenous formulations [12,13,14,15]. AMPs promote epidermal cell growth, repair the cortex and have anti-inflammatory properties [16,17], making AMPs a potential topical drug. Pexiganan and Omiganan are approved for topical application by the FDA, although they have not passed final clinical trials for a variety of reasons [18]. It is highly and widely expected that topical use of AMPs for wounds and skin infections might be the main route of their use, with their final success being especially in clinical practice regarding the treatment of pet skin infections in the near future. It is known to all that the pharmaceutical merits of AMPs make it the most suitable and special for treatment of skin infections via a clever style of merging its negative effects during systemic administration into positive ones during topical usage (Figure 1). Therefore, AMPs have become a hot spot in medical research and are expected to become a new method for treating antimicrobial infection and also representing a new method for the treatment of drug-resistant bacteria. AMPs have been reported as potential therapeutic topical agents in cases of canine pyoderma associated with staphylococcal resistance [19]. NZ2114 is an effective bactericide for Staphylococcus, especially methicillin-resistant Staphylococcus [20]. Transdermal drug delivery can overcome the skin barrier, effectively transfer active molecules to the treatment site and improve the bioavailability of drugs [21,22,23], which is an effective way to use AMPs in the treatment of canine skin diseases.

Water-soluble drugs, such as AMPs, face difficulty in penetrating the skin lipid layer. Therefore, osmotic agents are required to promote their transdermal delivery. Common methods for skin penetration include chemical permeation enhancers (CPEs), iontophoresis, electroporation, microneedles, ultrasound, magneto-swimming and photomechanical waves [24,25]. CPEs could reversibly reduce skin barrier function by destroying intercellular cuticle lipids; their safety and low-cost properties have made them the most commonly used transdermal method in pharmaceutics [26]. The CPEs most commonly used in pharmaceuticals include pyrrolidone, Azone, propylene glycol, lauric acid and oleic acid [27], which have relatively good penetration-promoting effects on water-soluble drugs. Pyrrolidone facilitates drug distribution in the lipid layer by disrupting or dissolving the lipid layer between cuticle cells [28,29]. Azone molecules can disrupt the arrangement of lipids in the lipid bilayer by forming a soup-spoon bending scheme, creating a permeable channel by disrupting the hydrogen bonds between ceramides [30]. Propylene glycol (PG) can improve the solubility of drugs in the carrier, increase keratin moisture and promote skin hydration [27,31]. Oleic acid (OA) and lauric acid (LA) can extract lipids, form new permeable regions in the lipid layer and form lipid-soluble ion pairs with basic drugs [32,33]. Therefore, different drugs require the selection of appropriate CPEs.

In this study, we conducted drug sensitivity tests on *S. pseudintermedius* isolated from canine skin lesions and studied the antibacterial activity of NZ2114 against *S. pseudintermedius* in vitro. NZ2114 is a derived AMP from the first fungal defensin, with specificity and effective activity towards *Staphylococcus* and *Streptococcus,* and its spray would be prepared by adding suitable chemical osmotic enhancer to study the in vitro transdermal permeability and to verify the therapeutic effect on a mouse model of superficial pyoderma infected with *S. pseudintermedius*, establishing the foundation for the topical administration of NZ2114 in the treatment of wounds or skin infections.

## 2. Results

### 2.1. Minimum Inhibitory Concentrations (MICs) of NZ2114 and Antibiotics against S. pseudintermedius

As shown in Table 1, NZ2114 showed a potent antibacterial effect against *S. pseudintermedius*, and its antibacterial activity was better than that of the antibiotic control groups. The MIC values of NZ2114 against *S. pseudintermedius* were 0.23–0.45 μM, which were slightly lower than those of Mupirocin (0.50 µM) and significantly lower than those of ofloxacin (1.38–88.55 μM) and lincomycin (2.26–9.03 μM). Additionally, lincomycin did not display any activity against *S. pseudintermedius* CGMCC 1.90002 and *S. pseudintermedius* CGMCC 1.90004.

### 2.2. Drug Sensitivity of S. pseudintermedius

The drug sensitivity of *S. pseudintermedius* to the following 22 antibiotics was determined by the disk diffusion method. The results are shown in Table 2. *S. pseudintermedius* CGMCC 1.90001 was resistant to penicillin, clindamycin, lincomycin and trimethoprim, and its sensitivity to ampicillin was at the intermediate value, while *S. pseudintermedius* CGMCC 1.90002 was resistant to penicillin, ampicillin, kanamycin, azithromycin, erythromycin, tetracycline, ciprofloxacin, ofloxacin, clindamycin, lincomycin and trimethoprim and moderately sensitive to ceftriaxone, which indicated that it had extensive drug resistance. *S. pseudintermedius* CGMCC 1.90003 was resistant to trimethoprim, and the sensitivity value to erythromycin, nitrofurantoin and clindamycin was mediated. Additionally, *S. pseudintermedius* CGMCC 1.90004 was resistant to kanamycin, azithromycin, erythromycin, clindamycin, lincomycin and trimethoprim and sensitive to the other antibiotics. In addition, *S. pseudintermedius* CGMCC 1.90005 was resistant to penicillin, ampicillin, kanamycin, azithromycin, erythromycin, tetracycline, clindamycin, lincomycin, trimethoprim and chloramphenicol and moderately sensitive to nitrofurantoin. 

### 2.3. Effect of Formulations on Activity of NZ2114

The effect of CPEs on the activity of NZ2114 was assessed by inhibition zone. As is shown in Table 3, Azone, N-methyl pyrrolidone (NMP) and PG could maintain the antibacterial activity of NZ2114, while LA and OA inhibited the antibacterial activity of NZ2114 (Table 3). As can be seen from the inhibition zone diameter in Table 3, an addition of 1% Azone significantly increased the antimicrobial activity of NZ2114, although different concentrations of Azone showed some antimicrobial properties. Both 10% and 20% PG have a similar effect in promoting the antimicrobial activity of NZ2114. However, the high concentration of PG could be irritating to the skin; therefore, 10% PG is the optimal choice. To optimize the osmotic effect of NMP, it is recommended to use 5% NMP, although 1% NMP has a greater ability to promote antimicrobial activity. PG is commonly used as an osmotic enhancer. However, its effect is not significant when used alone, so we chose 1% Azone + 10% PG and 5% NMP + 10% PG to prepare two kinds of NZ2114 sprays for subsequent experiments, respectively.

### 2.4. Penetrating Effect of NZ2114 Spray on Mouse Skin

**Figure 2:**
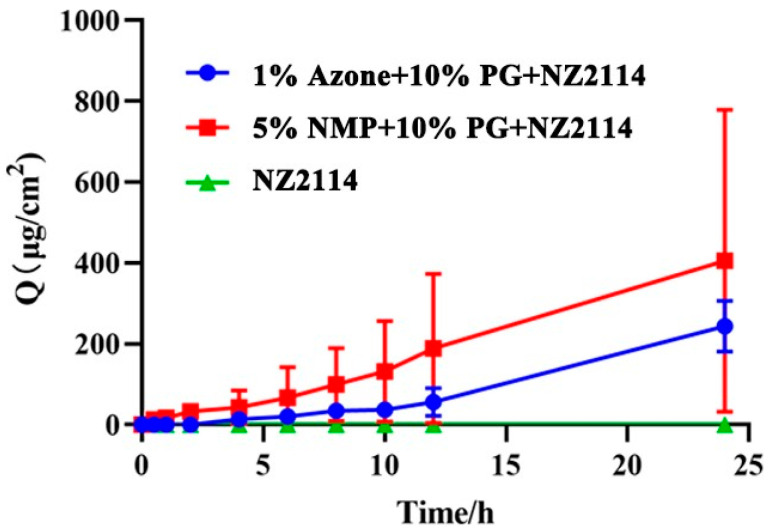
The cumulative permeation curve of NZ2114.

The addition of the permeability enhancers Azone, PG and NMP can increase the in vitro percutaneous permeability of NZ2114, and the cumulative permeability curves are shown in Figure 2. After 24 h in vitro osmosis, the cumulative osmosis of 1% Azone + 10% PG + NZ2114 and 5% NMP + 10% PG + NZ2114 was 244.149 and 405.245 μg/cm^2^, respectively, and the transdermal permeation rates were 10.173 and 16.885 µg/cm^2^/h, respectively. There was no NZ2114 detected in the receiving pool of the no osmotic agent added group.

### 2.5. Antimicrobial Effect of NZ2114 Spray on Superficial Pyoderma in Mice

**Figure 3:**
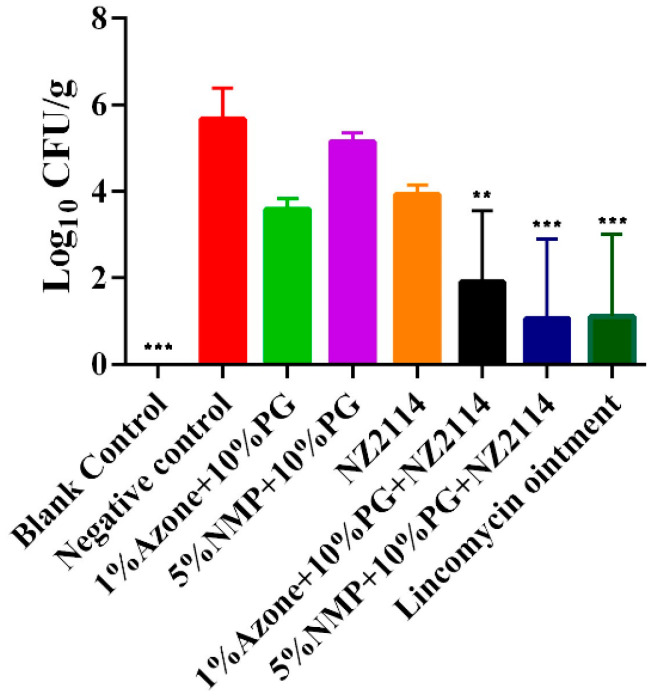
Microbiological analysis on day 5 after the treatment with NZ2114 spray. Lincomycin vitamin B6 ointment was used as the positive control (*n* = 3, mean ± SD). “** and ***” indicates the significant difference between negative control and treatment groups (** *p* < 0.01, *** *p* < 0.001).

As shown in Figure 3, the NZ2114 spray could effectively reduce the bacterial load in the skin lesions area. Compared with the negative control, 5% NMP + 10% PG NZ2114 significantly decreased in colony count to 1.059 ± 1.834 Log_10_ CFU/g, followed by the lincomycin VB6 ointment (1.100 ± 1.906 Log_10_ CFU/g) and 1%Azone + 10%PG + NZ2114 (1.900 ± 1.652 Log_10_ CFU/g). The results indicated that 5%NMP + 10%PG + NZ2114 had the best therapeutic effect against superficial pyoderma.

### 2.6. Effects of NZ2114 Sprays on Skin Irritation in Mice

As shown in Table 4, 1% Azone + 10% PG + NZ2114 and 5% NMP + 10% PG + NZ2114 (the final concentration of NZ2114 was 5 mg/mL) and their formulations had no irritating effect on intact or damaged skin in mice, which was similar to the negative control group of normal saline. The positive control group (30% SDS) had no irritating effect on intact skin in a single dose, but had a significant irritating effect on damaged skin and had an irritating effect on both intact and damaged skin in multiple doses, with erythema, edema and rough skin at the site of administration.

## 3. Discussion

The rapid increase in *S. pseudintermedius* multidrug and methicillin resistance has led to widespread interest in topical formulations for the treatment of canine skin diseases [34,35,36]. The results of isolation from 358 dog samples by Rana et al. showed that the prevalence of *S. pseudintermedius* in dogs was 45.3%, and the *S. pseudintermedius* isolates were resistant to more than three antimicrobial drug classes, with up to 91% and 84.7% resistance to nalidixic acid and erythromycin, respectively [37]. In this study, we analyzed the drug sensitivity of five strains of *S. pseudintermedius* isolated from canine skin lesions. The results showed that *S. pseudintermedius* CGMCC 1.90002 had the most extensive drug resistance, with 11 antimicrobial drug classes, followed by *S. pseudintermedius* CGMCC 1.90005 (10), *S. pseudintermedius* CGMCC 1.90004 (6), *S. pseudintermedius* CGMCC 1.90001 (4) and *S. pseudintermedius* CGMCC 1.90003 (1) (Table 2). All five *S. pseudintermedius* bacteria were resistant to Trimethoprim (SXT) and displayed Intermediate/Resistance to Clindamycin (CLI) and Lincomycin (LM). Compared with the other four strains, *S. pseudintermedius* CGMCC 1.90003 was more sensitive to antibiotics. It was noteworthy that NZ2114 had good antibacterial activity against these five *S. pseudintermedius* (0.23 µM~0.45 µM), especially the multidrug-resistant *S. pseudintermedius* 1.90002 (MIC: 0.23 µM), and its antibacterial activity was better than those of mupirocin, ofloxacin and lincomycin (0.5 µM ~> 288.95 µM) (Table 1). This indicates that NZ2114 could be used as a potential antibacterial agent for *S. pseudintermedius*.

For the treatment of skin diseases, traditional oral drug delivery requires first-pass metabolism in the liver and breakdown of active substances in the gastrointestinal tract, resulting in low oral bioavailability of some drugs, and it is difficult for the drugs to play a therapeutic role when they reach the focal points of skin diseases [38,39]. Transdermal drug delivery systems offer significant advantages over oral drug delivery [40,41,42]; the transdermal dose required for the therapeutic effect of a drug is lower than the oral dose because the transdermal administration avoids the first-pass metabolism in the liver [43]. However, due to the barrier effect of the skin’s stratum corneum (SC), which prevents some large molecule drugs (molecular weight greater than 500 Daltons) from penetrating the SC [44], only 1–5% of drugs are able to penetrate the skin, resulting in low bioavailability of large molecule drugs [43,45]. Therefore, transdermal delivery of hydrophilic peptides remains a challenge due to their poor cellular absorption and transdermal permeability [46]. In this study, in order to increase the transdermal penetration of the antimicrobial peptide NZ2114, five common CPEs were selected and their effects on the antibacterial activity of NZ2114 were studied. The findings revealed that Azone, PG and NMP have no influence on the antibacterial activity of NZ2114, whereas OA and LA impeded the activity of NZ2114 even at a low concentration (1%) (Table 3). This may be attributed to the fact that these two substances are anionic surfactants with strong electrostatic attraction towards the cationic antimicrobial peptide, hindering the interaction between NZ2114 and bacteria, consequently reducing the antimicrobial efficacy [47]. As a highly effective, safe and non-toxic CPE, the added amount of Azone is generally 0.2–2%. Due to the antibacterial activity of Azone itself, the added amount of 1% Azone was chosen according to the results of the antibacterial zone, which could better reflect the antibacterial property of NZ2114 (Table 3). NMP is a highly effective CPE among FDA inactive ingredients and has no antimicrobial activity of its own. While the permeation effect of PG may not be as potent as those of Azone and NMP when it is used alone, it even blocks drug penetration, as high concentrations of PG increase viscosity and hinder drug diffusion [48]. When combined with other CPEs, PG can not only increase the solubility of drugs and CPEs but also play a synergistic role [49]. Thus, research on the binary enhancer combination of PG and Azone/NMP is warranted, and a relatively low concentration of PG (10%) was selected. Based on that, the two different NZ2114 sprays were prepared by mixing 1% Azone and 10% PG with NZ2114 or 5% NMP and 10% PG with NZ2114. The results of in vitro transdermal penetration showed that 5% NMP + 10% PG significantly enhanced the penetration effect of NZ2114, which was 1.66-fold higher than that of 1% Azone + 10% PG. Although some studies have proved that the osmotic effect of Azone is stronger than that of NMP [48,50], the transdermal effect of different substances cannot be generalized, and different proportions, combined effects and the properties of the drug itself will influence the effect of transdermal agents.

NZ2114 had strong antibacterial activity against Gram-positive bacteria in vitro; however, the low transdermal penetration directly affects its effectiveness as a topical drug. Its combination with CPEs could significantly enhance the transdermal penetration effect to reduce the bacterial load of superficial pyoderma in mice infected with *S. pseudintermedius*. The effect of 5% NMP + 10% PG + NZ2114 was better than those of 1% Azone + 10% PG + NZ2114 and the positive control Lincomycin vitamin B6, which is attributed to the fact that NZ2114 could penetrate the skin barrier effectively with 5% NMP + 10% PG. In addition, the two NZ2114 sprays had no irritating effect on the mouse skin, laying the foundation for the development of an effective and non-toxic topical product which conforms to the requirement that external preparations have no irritation to the skin [51]. Although NZ2114 sprays have shown a positive therapeutic effect on superficial pyoderma in mice, further verification is required to determine its efficacy in the target animal (canine). In addition, the long-term safety of the spray needs to be assessed, which will be the focus of our next study.

## 4. Materials and Methods

### 4.1. Strains, Reagents and Mice

The test strains of *S. pseudintermedius* CGMCC 1.90001, *S. pseudintermedius* CGMCC 1.90002, *S. pseudintermedius* CGMCC 1.90003, *S. pseudintermedius* CGMCC 1.90004 and *S. pseudintermedius* CGMCC 1.90005 isolated from canine skin lesions were obtained from the China Agricultural University, which were stored in the China General Microbiological Culture Collection Center (CGMCC). The quality control strains of *Staphylococcus aureus* (*S. aureus*) ATCC 25923 and *S. aureus* ATCC 29213 were purchased from the American Typical Strain Collection Center (ATCC).

NZ2114 was expressed by *P. pastoris* X-33 in our lab and purified based on our previous protocol (with purity over 90%) [20]. Yeast extract and tryptone were purchased from Thermo Fisher Scientific (Shanghai, China), Azone from Hubei Kejie Pharmaceutical Co., Ltd. (Wuhan, China), N-methyl pyrrolidone (NMP) and oleic acid (OA) from Macklin Biochemical Technology Co., Ltd. (Shanghai, China) and propylene glycol (PG) and lauric acid (LA) from Aladdin reagent (Shanghai) Co., Ltd. (Shanghai, China). Lincomycin and ofloxacin were purchased from Meilun Biotechnology Co., Ltd. (Dalian, China). Mupirocin was purchased from Yuanye Biotechnology Co., Ltd. (Shanghai, China) and mannitol sodium chloride agar culture medium was purchased from Qingdao hi tech Industrial Park Haibo Biotechnology Co., Ltd. (Qingdao, China). Lincomycin B6 ointment was purchased from Heilongjiang Tianchen Pharmaceutical Co., Ltd. (Harbin, China) and antibiotic-sensitive pieces were purchased from Wenzhou Kangtai Biological Technology Co., Ltd. (Wenzhou, China). The formulations of NZ2114 used in this manuscript are shown in Section 2.3 (3rd paragraph of the result). All other chemical reagents were analytical grade and biological reagents were used as received.

Female BALB/c mice aged 6–8 weeks and 15 g ICR mice (SPF) were purchased from Beijing Vital River Laboratory Animal Technology Co., Ltd. (Beijing, China). All animal studies were approved by the experimental animal ethics committee and its inspection committee (AEC-CAAS-20090609) of the Feed Research Institute of the Chinese Academy of Agricultural Sciences in accordance with the provisions of the animal protection and use Committee of the Feed Research Institute of the Chinese Academy of Agricultural Sciences.

### 4.2. MICs of NZ2114 against S. pseudointermedia

The MICs of NZ2114 against *S. pseudointermedia* were determined by the microtiter dilution method to evaluate its antibacterial activity [52]. The mid-logarithmic phase bacteria were diluted to 1 × 10^5^ CFU/mL with fresh Luria–Bertani (LB) medium; a volume of 90 μL of bacterial suspension and 10 μL of serially two-fold-diluted peptide with final concentrations of 0.0625–128 μg/mL were added into 96-well plates. The plates were incubated for 16–18 h at 37 °C. The MIC values were determined as the lowest peptide concentration at which no bacterial growth was observed. Ofloxacin, mupirocin and lincomycin were used as controls. All assays were performed in triplicate.

### 4.3. S. pseudintermedius Susceptibility Assay

According to the standards implemented by the clinical and Laboratory Standards Institute (CLSI) [53], the drug sensitivity of *S. pseudintermedius* to common antibiotics was determined by the disk diffusion method [54,55]. The specific steps are as follows: *S. pseudintermedius* was cultured to the logarithmic phase of growth and diluted to 0.5 Mcfarland Standard with Mueller–Hinton broth (MHB) medium. A volume of 250 μL bacterial suspension was evenly coated on the Mueller–Hinton agar (MHA) with a coating rod. After the surface of the medium was dried, the drug sensitive tablets were pasted on the agar plate with sterile tweezers. Each plate was placed with two kinds of drug sensitive tablets (placed symmetrically). The spacing between the two drug sensitive tablets was greater than 24 mm, and the distance between the drug sensitive tablets and the edge of the plate was greater than 15 mm. The drug sensitive tablets were gently pressed to prevent them from falling off. After 18 h of inverted culture in a 37 °C constant temperature incubator, the diameter of the inhibition zone was measured with each drug sensitive tablet against *S. pseudointermedia* (expressed in mm). *S. aureus* ATCC 25923 and *S. aureus* ATCC 29213 were used as control strains. All assays were performed in triplicate.

### 4.4. Formulation Screening for NZ2114 Spray

The inhibition zone assay was used to analyze the effect of different CPEs on the antibacterial activity of NZ2114. The different concentrations of Azone (0.5%, 1% and 2%), PG (5%, 10% and 20%), OA (1%, 5% and 10%), NMP (1% and 5%) and LA (1%, 3% and 5%) were chosen to test their effects so that the suitable or optimized combination could be screened. For the NZ2114 sprays preparation, the CPEs were mixed with water, and then the equivalent mass of NZ2114 was dissolved in the above mixture and filtered through a 0.22 μm sterile membrane to remove bacteria for the subsequent experiments. *S. pseudointermedius* CGMCC 1.90002 was cultured in LB medium to mid-logarithmic phase and diluted to 1 × 10^6^ CFU/mL with LB agar medium. The samples were prepared using formulations of different concentrations and with a final concentration of 50 μg/mL NZ2114. A volume of 30 μL NZ2114 spray was dropped by pipette onto LB agar plate, and the diameter of inhibition zone was measured after overnight incubation. NZ2114 solution in water at equal concentration was used as the positive control, while formulations without NZ2114 were used as the blank controls. All assays were performed in triplicate.

### 4.5. In Vitro Skin Penetration Experiments

The 6~8 weeks old female BALB/c mice were killed by humanitarian anesthesia. The back skin was depilated with hair removal cream and cut, the subcutaneous adipocytes and fascia were stripped and the skin was cleaned with isotonic normal saline until clarification. The mice skin was placed in a centrifugal tube with normal saline and then stored in a refrigerator at 4 °C for 3 days. Before the experiment, the skin of mice was prebalanced in normal saline at 25 °C.

The in vitro transdermal permeability of antimicrobial peptides was tested by the Franz diffusion cell method [56]. Skin specimens were sandwiched securely between the donor and receptor compartment of each Franz diffusion cell, and the transdermal diffusion of the effective diameter was 1.8 cm. The supply pool contained 1 mL NZ2114 spray (3.4 mg/mL), the receiving pool was filled with saline solution (6.5 mL) and the skin fully was immersed in saline. The supply pool was sealed with a sealing membrane to prevent moisture evaporation, and the receiving pool was maintained with constant-speed mixing and a temperature of 37 °C at a rotation rate of 400 rpm/min. Samples with a volume of 1 mL in the receiving pool were collected at 0, 0.5, 1, 2, 3, 4, 6, 8, 10, 12 and 24 h, respectively, and the same amount of normal saline at 37 °C was immediately supplemented. The collected samples were centrifuged at 12,000 rpm/min for 5 min, filtered with 0.22 µm membrane and determined by high-performance liquid chromatography (HPLC). The Zorbax eclipsc plus C18 (4.6 × 250 mm, 5 μm) chromatographic column was used for HPLC. The mobile phase was 0.1% trifluoroacetic acid acetonitrile and 0.1% trifluoroacetic acid water, the flow rate was 1 mL/min, the column temperature was 35 °C and the detection wavelength was 280 nm. The results were expressed in terms of the cumulative amount of NZ2114 penetrated into the skin. NZ2114 aqueous solution was used as a blank control. Three replicates were performed. The following equation was used to calculate the cumulative drug release Q:

$$Q=\sum_{t=1}^{n}Ci\left(i=1,\;2,\;3,\ldots \right)$$

### 4.6. Superficial Pyoderma Assay

The 24 female BALB/c mice (6~8 weeks old) were randomly divided into 8 groups (3 mice/group), which included blank control, negative control, positive control (lincomycin ointment), 1% Azone + 10% PG group, 5% NMP + 10% PG group, NZ2114 group, 1% Azone + 10% PG + NZ2114 group and 5% NMP + 10% PG + NZ2114 group. The mice were depilated with depilatory cream 48 h before the experiment. The cuticle of the skin was removed by the tape striping (TAP) method, and the specific steps are as follows: The back of the mice was avulsed 10 times with equal force of medical tape 1 × 2 cm^2^ in size to remove the cuticle, and a 1 mm micro needle roller was rolled once on the back from top to bottom, from left to right without pressure. Then, the back of mice was infected with 10^7^ CFU/mL of *S. pseudintermedius* CGMCC 1.90002 (50 μL) to construct the model of superficial pyoderma. After infection for 2 h, the mice were treated with NZ2114 spray twice a day at the concentration of 2 mg/mL by pipette. The lincomycin vitamin B6 ointment treatment group (3.4 mg/mL) served as positive control [4,57]. After 5 days of continuous treatment, mice were killed by humanitarian anesthesia, the infected area of the skin was collected and weighed. Then, 100 μL of saline was added to each 0.1 g of skin for tissue homogenization. The homogenate was coated onto LB plates, and the colonies were counted after 18 h of incubation in incubator at 37 °C. The number of colonies in the treated groups were compared to the untreated group (negative control) to determine the reduction in skin bacteria.

### 4.7. Skin Irritation Assay

The skin irritation assay consisted of a single-dose and multiple-dose skin irritation assay. The difference was that the multiple-dose skin irritation test measures skin symptoms after 7 days of continuous administration. The ICR mice (SPF, weight: 15 g) were divided into intact skin group and damaged skin group. The back of the mice was depilated with depilatory cream before administration, and each area was about 1.0 × 1.0 cm^2^. In the damaged skin group, the depilated part was disinfected, and the skin was rubbed with sandpaper until bleeding. The 100 µL of NZ2114 spray was applied to the administration site with a final concentration of 5 mg/mL by pipette. The normal saline group and 30% sodium dodecyl sulfate (SDS) group were used as the negative control and the positive control, respectively. The spray formulations without NZ2114 served as the blank control. The residual drugs were washed off with warm water after administration at 1, 24, 48 and 72 h. The intensity of stimulation was judged according to the skin stimulation response scale [58] and it was recorded whether there was erythema or edema at the administration site.

### 4.8. Statistical Analysis

All data from at least three independent tests were expressed as the mean ± SD. One-way ANOVA (GraphPad Prism 8.0 software, San Diego, CA, USA) was used for statistical analysis. A *p* value less than 0.05 was considered to indicate a significant difference (* *p* < 0.05, ** *p* < 0.01, *** *p* < 0.001, **** *p* < 0.0001).

## 5. Conclusions

In this study, it is demonstrated that topical NZ2114 formulations prepared via the incorporation of NZ2114 into CPEs promoted the NZ2114 transdermal penetration effect. The optimal formulation, 5% NMP + 10% PG + NZ2114, showed 405% greater skin permeability at 24 h, and the formulation could effectively kill *S. pseudintermedius* in superficial pyoderma in mice. Moreover, the topical NZ2114 formulations displayed good security. The findings indicate that the 5% NMP + 10% PG + NZ2114 formulation could be an effective alternative against superficial pyoderma. Due to the shortage of pet and livestock drugs, further studies should be conducted on the NZ2114 spray as a topical antimicrobial drug against skin infections in pets and livestock (such as canine pyoderma and sheep subcutaneous abscess) in order to meet the actual needs for clinical medication in pets and livestock and to improve their health. Due to its druggability, NZ2114 also has the potential to be developed as a topical formulation for human skin infections and wound healing. Therefore, topical application is the preferred direction for its clinical use in the future.

## Figures and Tables

**Table 1:** MICs of NZ2114 and antibiotics against *S. pseudintermedius*.

Strains	MIC							
	NZ2114 ^a^		Mupirocin		Ofloxacin		Lincomycin	
	μg/mL	μM	μg/mL	μM	μg/mL	μM	μg/mL	μM
*S. pseudintermedius* CGMCC 1.90001	2	0.45	0.25	0.50	0.5	1.38	1	2.26
*S. pseudintermedius* CGMCC 1.90002	1	0.23	0.25	0.50	32	88.55	>128	>288.95
*S. pseudintermedius* CGMCC 1.90003	2	0.45	0.25	0.50	0.5	1.38	1	2.26
*S. pseudintermedius* CGMCC 1.90004	2	0.45	0.25	0.50	1	2.77	>128	>288.95
*S. pseudintermedius* CGMCC 1.90005	2	0.45	0.25	0.50	1	2.77	4	9.03

Note: ^a^ The sequence of NZ2114 is GFGCNGPWSEDDIQCHNHCKSIKGYKGGYCARGGFVCKCY; Molecular weight: 4417 Da; pI: 8.62; Charge: +3; The grand average of hydropathicity (GRAVY): −0.672.

**Table 2:** The susceptibility analysis of *S. pseudintermedius*.

	*S. pseudintermedius* CGMCC 1.90001		*S. pseudintermedius* CGMCC 1.90002		*S. pseudintermedius* CGMCC 1.90003		*S. pseudintermedius* CGMCC 1.90004		*S. pseudintermedius* CGMCC 1.90005	
	D ^a^ (mm)	Susceptibility	D ^a^ (mm)	Susceptibility	D ^a^ (mm)	Susceptibility	D ^a^ (mm)	Susceptibility	D ^a^ (mm)	Susceptibility
Penicillin (PEN)	11.5	R	8.5	R	39.5	S	37.0	S	7.5	R
Ampicillin (AMP)	14.5	I	10.5	R	35.5	S	38.0	S	12.0	R
Cefatriaxone (CRO)	28.5	S	14.5	I	32.0	S	30.5	S	28.5	S
Cefazolin (CZO)	32.0	S	25.5	S	42.5	S	42.0	S	28.5	S
Cefotaxime (CTX)	36.5	S	23.5	S	42.5	S	37.5	S	33.5	S
Imipenem (IPM)	42.5	S	45.5	S	45.5	S	45.5	S	42.0	S
Cefoxitin (FOX)	35.5	S	32.0	S	30.5	S	34.5	S	33.5	S
Oxacillin (OXA)	27.0	S	25.5	S	29.5	S	36.0	S	22.5	S
Vancomycin (VAN)	15.5	S	17.3	S	14.5	S	17.0	S	17.5	S
Kanamycin (KAN)	23.5	S	9.5	R	23.0	S	7.0	R	12.0	R
Azithromycin (AZM)	22.0	S	6.0	R	23.5	S	6.0	R	6.0	R
Erythromycin (ERY)	25.5	S	6.0	R	22.0	I	6.0	R	7.0	R
Tetracycline (TCY)	20.5	S	9.3	R	22.5	S	21.0	S	8.0	R
Ciprofloxacin (CIP)	27.0	S	6.5	R	28.0	S	24.0	S	23.0	S
Ofloxacin (OFX)	24.8	S	6.0	R	24.5	S	22.0	S	20.5	S
Nitrofurantoin (NIT)	22.5	S	21.0	S	22.0	S	20.0	S	15.5	I
Clindamycin (CLI)	5	R	6.0	R	21.0	I	6.0	R	12.0	R
Lincomycin (LM)	5	R	6.0	R	19.0	I	6.0	R	10.0	R
Trimethoprim (SXT)	6	R	6.0	R	6.0	R	6.0	R	6.0	R
Chloramphenicol (CHL)	21	S	24.0	S	22.5	S	20.0	S	11.5	R
Rifampicin (RIF)	27.5	S	24.0	S	26.0	S	27.0	S	29.0	S
Linezolid (LNZ)	25	S	28.5	S	21.5	S	21.0	S	28.5	S

Note: D ^a^: Inhibition zone diameter; S: Sensitive, I: Intermediate, R: Resistance.

**Table 3:** Inhibition zone diameter of formations and NZ2114 sprays.

Inhibition Zone Diameter/mm														
	Azone (%)			PG (%)			OA (%)			NMP (%)		LA (%)		
	0.5	1	2	5	10	20	1	5	10	1	5	1	3	5
Positive Control	14.70 ± 0.67													
Blank Control	16.67 ± 0.58	17.17 ± 0.29	18.00 ± 1.00	0	0	0	0	0	0	0	0	0	0	0
NZ2114 sprays	17.17 ± 0.29	18.17 ± 0.76	18.23 ± 0.58	15.83 ± 1.04	16.33 ± 1.19	16.37 ± 0.58	0	0	0	15.17 ± 0.29	14.67 ± 0.58	0	0	0

**Table 4:** Results of skin irritation assay.

Administration	Groups	Test Substance	Total Score				Average			
			Time (h)							
			1	24	48	72	1	24	48	72
Single dose	Intact skin	NZ2114 sprays	0	0	0	0	0	0	0	0
		Blank Control	0	0	0	0	0	0	0	0
		Negative Control	0	0	0	0	0	0	0	0
		Positive Control	0	0	0	0	0	0	0	0
	Damaged skin	NZ2114 sprays	0	0	0	0	0	0	0	0
		Blank Control	0	0	0	0	0	0	0	0
		Negative Control	0	0	0	0	0	0	0	0
		Positive Control	8	7	7	6	2.7	2.3	2.3	2
Multiple doses	Intact skin	NZ2114 sprays	0	0	0	0	0	0	0	0
		Blank Control	0	0	0	0	0	0	0	0
		Negative Control	0	0	0	0	0	0	0	0
		Positive Control	9	9	9	9	3	3	3	3
	Damaged skin	NZ2114 sprays	0	0	0	0	0	0	0	0
		Blank Control	0	0	0	0	0	0	0	0
		Negative Control	0	0	0	0	0	0	0	0
		Positive Control	12	9	9	9	4	3	3	3

## Data Availability

All data generated or analyzed during this study are included in this published article.
